# A qualitative study of psychological, social and behavioral barriers to appropriate food portion size control

**DOI:** 10.1186/1479-5868-10-92

**Published:** 2013-08-01

**Authors:** Michelle Spence, M Barbara E Livingstone, Lynsey E Hollywood, Eileen R Gibney, Sinéad A O’Brien, L Kirsty Pourshahidi, Moira Dean

**Affiliations:** 1 Institute for Global Food Security, School of Biological Sciences, Queen’s University Belfast, Belfast, UK; 2 Northern Ireland Centre for Food and Health (NICHE), University of Ulster, Coleraine, UK; 3 Ulster Business School, University of Ulster, Coleraine, UK; 4 UCD Institute of Food and Health, School of Agriculture and Food Science, UCD, Dublin 4, Republic of Ireland

**Keywords:** Food portion size, Barriers, Obesity, Consumers, Qualitative study

## Abstract

**Background:**

Given the worldwide prevalence of overweight and obesity, there is a clear need for meaningful practical healthy eating advice - not only in relation to food choice, but also on appropriate food portion sizes. As the majority of portion size research to date has been overwhelmingly quantitative in design, there is a clear need to qualitatively explore consumers’ views in order to fully understand how food portion size decisions are made. Using qualitative methodology this present study aimed to explore consumers’ views about factors influencing their portion size selection and consumption and to identify barriers to appropriate portion size control.

**Methods:**

Ten focus groups with four to nine participants in each were formed with a total of 66 persons (aged 19–64 years) living on the island of Ireland. The semi-structured discussions elicited participants’ perceptions of suggested serving size guidance and explored the influence of personal, social and environmental factors on their food portion size consumption. Audiotapes of the discussions were professionally transcribed verbatim, loaded into NVivo 9, and analysed using an inductive thematic analysis procedure.

**Results:**

The rich descriptive data derived from participants highlight that unhealthy portion size behaviors emanate from various psychological, social and behavioral factors. These bypass reflective and deliberative control, and converge to constitute significant barriers to healthy portion size control. Seven significant barriers to healthy portion size control were apparent: (1) lack of clarity and irrelevance of suggested serving size guidance; (2) guiltless eating; (3) lack of self-control over food cues; (4) distracted eating; (5) social pressures; (6) emotional eating rewards; and (7) quantification habits ingrained from childhood.

**Conclusions:**

Portion size control strategies should empower consumers to overcome these effects so that the consumption of appropriate food portion sizes becomes automatic and habitual.

## Background

Overweight and obesity is a serious public health concern which is associated with an increased risk of adverse health outcomes, including type 2 diabetes, cardiovascular disease and certain cancers [1]. Numerous behavioral and environmental factors have been associated with obesity, including a decreased level of occupational and leisure time activity and a ready access to food [2].

While large cross-sectional dietary intake surveys have shown increases in food and beverage portions over the last 30 years [3,4], experimental studies have demonstrated the effects of varying food portion size on energy intake. In both laboratory and more naturalistic settings (i.e. a cafeteria-style restaurant), increasing the portion size served has been shown to positively influence energy intake at single eating occasions [5-12] and over the course of one to 28 days [13-17]. This effect has been demonstrated with amorphous food [8], packaged snacks [10], unpackaged snacks [18], beverages [19], and, single meals [6] with both a high [5] and low [12] energy density. The effect is particularly pronounced with energy dense foods [6] and even holds true when the food is rated unfavourable with regards to taste, e.g. stale popcorn [11]. The tendency to consume more energy when served larger portions has been observed regardless of demographic characteristics such as socioeconomic status [20], age [9], BMI [9,21] and sex [8,9], and, also occurs when individuals eat alone [10] as well as in company [12]. In sum, research implies that consumers have a tendency to eat all that’s served, irrespective of the amount.

In a review of the literature, Steenhuis and Vermeer (2009) [22] attribute the self-selection and consumption of larger portions to a combination of ‘portion distortion’ (i.e. perceiving larger portions as appropriate amounts of food to consume at one time), ‘value for money’ and an ‘inability to accurately monitor food intake’. Larger packages in supermarkets, larger servings in restaurants, larger tableware in homes, and eating distractions are only some of the factors within the food and eating environment that have contributed to portion size distortion, and thereby eliciting overeating [23]. Additional research has highlighted the perverse effects of small packages on consumption self-regulation [24,25]; confirmed the positive association between the ‘perceived healthiness of a food’ and consumption quantities [26]; and established a role for expected satiety/satiation as a predictor of meal size [27].

As the majority of portion size research to date has been overwhelmingly quantitative in design (i.e. experimental, descriptive, and correlational), there is a clear need to complement this with a qualitative exploration of consumers’ views in order fully understand how food portion size decisions are made. Suter states: *‘To understand a complex phenomenon, you must consider the multiple “realities” experienced by the participants themselves—the “insider” perspectives’*[28]. An informed appreciation of these psychological, social and behavioral influences from the *“insider”/*consumer perspective will empower health professionals to design more pragmatic obesity prevention and treatment interventions that support appropriate portion size control. Therefore, the aim of the present study was to qualitatively explore consumers’ views about factors influencing their portion size selection and consumption and to identify barriers to appropriate portion size control.

For the purposes of this paper the term ‘portion size’ will be used to denote the amount of food an individual consumes at a single eating occasion (e.g. dinner, snack), while the term ‘serving size’ will denote the quantity recommended to be consumed on one eating occasion, as defined by a manufacturer or in dietary guidance.

## Methods

### Focus group recruitment

Male and female participants were recruited using a purposive sampling method at community centers, churches and universities, and were invited to attend one of ten scheduled focus groups sessions (April to June 2011). Every effort was made to include participants from different age groups and educational backgrounds, and from city, urban and rural locations to capture a range of views. Groups were segregated on the basis of sex as it was thought this might facilitate easier interaction between group members. Seventy-two consumers were recruited, of whom sixty-six took part in the focus groups. Five withdrew from the study on the day (too busy or unwell), and a further one did not attend their assigned focus group. Of the ten focus group discussions, three were held in the Republic of Ireland (ROI) (n=2 female groups; n=1 male group), and seven where held in Northern Ireland (NI) (n=4 female groups; n=3 male groups). Each group consisted of four to nine participants (Table 1). This study was conducted according to the guidelines laid down in the Declaration of Helsinki and all procedures involving human subjects were approved by the Queens University Belfast Ethical Committee. Written informed consent was obtained from all subjects.

**Table 1:** Focus group demographics

**Group**	**Location**	**Gender**	**Age range (years)**	**Number of participants**
One	ROI	Female	20-29	8
Two	ROI	Female	30-40	9
Three	ROI	Male	29-39	8
Four	NI	Male	29-59	6
Five*	NI	Male	20-25	7
Six	NI	Female	21-59	4
Seven	NI	Male	19-24	8
Eight†	NI	Female	39-45	4
Nine	NI	Female	22-63	5
Ten‡	NI	Female	35-64	7

Abbreviations: *ROI*: Republic of Ireland, *NI*: Northern Ireland.

*All participants were recruited from a rugby team.

†All participants were recruited from a parent and toddler group for children of pre-school age.

‡All participants were recruited from a slimming club.

### Focus group procedures

Focus group discussions were facilitated by an experienced moderator (MS) in an informal setting convenient to subjects. As an ice-breaker, participants were asked to introduce themselves on a first name basis and describe what they ate for dinner the previous evening. The moderator then gave instruction on ground rules (e.g. not talking over each other, the importance of confidentiality) before proceeding to a series of guided open-ended topics (Table 2). As the final closing question, participants were asked to recall their previous evening meal and identify factors which influenced their portion size consumption. The moderator sought input from all participants and encouraged elaboration on the issues, emphasising that there were ‘no right or wrong answers’, and, that all views were valid, even if entirely different. Each focus group session was audio recorded and lasted between 50 and 90 minutes. Additional self-reported demographic and household characteristics of the participants were obtained via a brief end-of-session questionnaire. Subsequently, participants were thanked and were informed that they could speak to any member of the research team about any issues raised. An honorarium of £20 (NI residents) or €25 (ROI residents) was paid for time and travel costs of study participation.

**Table 2:** Outline of the moderator’s questioning route

**Question category**	**Question**
Opening questions	Please introduce yourself and describe what you ate for dinner the previous evening.
Introductory questions	What does a healthy diet mean for an adult?
	How do you know how much food you should eat?
Key questions in regard to consumer understanding of food portions	What does the term portion size mean to you?
	Do you ever attempt to control food portion sizes, and if so, how and why is this done?
Key questions in regard to consumer awareness and use of portion size guidance	Where do you find information/guidance on food portion size?
	What is the nature of the advice and how helpful is it? What is your overall impression of the Food Pyramid used in Ireland and the UK ‘Eatwell Plate’?
	Do you want further information about food portions, and if so, who from and in what format?
Key questions in regard to food purchase	Does food packaging/price influence how much you eat?
Key questions in regard to the selection of portion size prior to consumption	When preparing food, how do you determine the right proportion of food for yourself? (e.g. measuring).
	Are different quantities of food served to different household members?
	Does the amount served at one mealtime depend on other foods eaten throughout the day?
Key questions in regard to portion size decisions during consumption	Once a meal has been served, what factors influence how much you consume?
	Are second servings often consumed, and if so, why? Who serves them?
Key questions in regard to the eating environment	Does eating at different places or even with different people influence the amount you eat? If so, how?
	Do any other places or times influence the amount you eat?
Ending questions	Can you think of any other instances, whereby, your portion size would change?
	What factors influenced how much you ate last night?

### Focus group questions

The moderator’s questioning route (Table 2) was designed following a literature review of factors influencing food portion size consumption. Prompts were used when necessary to redirect or facilitate discussion. Questions were pretested for clarity and comprehension with a discrete mixed gender sample of the target group (n=8, age range 25–36 years) and refined prior to implementation. The seven main topic areas were designed to elicit participants’ perceptions on suggested serving size guidance, and, to explore antecedents and mediators to food portion size consumption. The use of food guide models, namely, the ‘Eatwell Plate’ [29] and Food Pyramid [30] (used in NI and the ROI, respectively), were employed to gauge consumer perception, stimulate discussion, and, focus the discussion unto the concept of food portion sizes.

### Analysis of focus group transcripts

Focus groups were professionally transcribed verbatim, reviewed by the moderator for accuracy, and, imported into the qualitative data analysis software package NVivo 9 (QSR International Pty Ltd, Doncaster, Victoria, Australia). An inductive thematic analysis procedure, as outlined by Braun and Clarke [31], was applied to the data in order to identify themes. Initially, transcripts were read repeatedly in order to achieve data ‘immersion’ and generate initial ideas about what was interesting in the data. Subsequently, barriers which hindered or prevented appropriate food portion size control were systematically coded across the entire dataset. The third and fourth phases involved grouping the codes (and their related data) into potential themes and then inspecting these themes for overlap and commonalities and, where necessary, refining themes (i.e. collapsing or dividing themes). This refinement ensured that data within themes formed a coherent pattern and that there were ‘clear and identifiable distinctions’ between themes [31]. The transcripts were also re-read at this stage to ensure that no data within themes had been missed in the earlier coding stages.

A peer-review process was used throughout data analysis. To begin with, three authors (MS, a nutritionist; MD, a psychologist; LKP, a nutritionist) and one outside researcher (a nutritionist) independently coded the same randomly selected transcripts (n=3) and discussed the codes to verify the validity and reliability of their application to the data. MS and MD then independently coded the remaining transcripts, and grouped the codes into themes appearing across all ten groups Through discussions, MS and MD reached a consensus on the assignment of all themes (inter-rater reliability was therefore equal to 1.00 as there was full agreement) and extracted quotations to illustrate typical views within each theme. Care was taken to ensure that the codes which were identified in some focus groups were not generalised to all groups under the interpretation of the identified themes. Both reviewers (MS and MD) agreed that data saturation had occurred as no new codes emerged from the final three focus group transcripts. Socio-demographic data was summarised using IBM SPSS Statistics version 19 software (SPSS, Chicago, IL).

## Results

### Participant characteristics

The characteristics of the 66 consumers who participated are shown in Table 3. Mean (SD) age was 34.2 (12.3) years.

**Table 3:** Characteristics of focus group participants

**Characteristic**	**N=66**	
	***n***	**%**
Gender		
Female	37	56
Male	29	44
Living arrangements		
Lives alone	10	15
Lives with partner	11	17
Lives with partner and child(ren)	16	24
Lives with children	1	2
Lives with parents	1	2
Lives with parents and sibling(s)	11	17
Lives with other adults*	16	24
Occupation status		
Employed full-time/part-time	33	50
Full-time homemaker	7	11
Unemployed	3	5
Student	22	33
Retired	1	2
Highest level of education		
Basic school	6	9
A-level	17	26
Vocational training	11	17
University level	32	48
Special diet		
Weight reducing diet	13	20
Diabetic diet	3	5
Renal diet	1	2
Vegetarian diet	5	11
Vegan diet	2	3
No special diet	42	64
BMI† (kg/m^2^)		
Less than 18.5 (underweight)	3	5
18.5 to less than 25 (normal)	20	30
25 to less than 30 (overweight)	21	32
30 or more (obese, excluding morbidity)	15	23
40 or more (morbidly obese)	6	9
Unknown	1	1

*Comprised of participants living with relatives (6%) and non-relatives (94%) not falling into another category.

†BMI based on self-reported height and body weight.

### Barriers to the practice of appropriate portion size control

Seven key interacting barriers to appropriate portion size control were identified: (1) lack of clarity and irrelevance of suggested serving size guidance; (2) guiltless eating; (3) lack of self-control over food cues; (4) distracted eating; (5) social pressures; (6) emotional eating rewards; and (7) quantification habits ingrained from childhood.

#### ***(1) Lack of clarity and irrelevance of suggested serving size guidance***

During the discussion, it became evident that consumers felt they were repeatedly exposed to numerous conflicting, inconsistent (e.g. *“they’re always changing their minds”*) and confusing messages in relation to ‘what’ and ‘how much’ they should eat. Across all groups, consumers recalled different suggested serving sizes for particular foods and food groups, gleaned from the public, private, and voluntary sectors (e.g. dietitians, slimming groups, food packets)*.* More often than not, this mix of advice contributed to its reduced effectiveness:

Group nine participant: *“…you’re permeated with this stuff [healthy eating guidance]…you’re so bogged down with it, it’s actually information overload and it just gets to the point where it’s not effective anymore.”*

Responses also highlighted the difficulty of obtaining serving guidance in meaningful amounts which could be easily remembered. Specifically, the majority of participants acknowledged that household measurements were open to interpretation (e.g. *“a glass could be a shot glass or pint glass”*), and, they poorly recognised the actual quantity of servings specified in imperial (e.g. 2oz cheese) and metric measurements (e.g. 56g cheese):

Group nine participant: *“But to me, it’s like talking about a trillion pounds; I actually have no idea what that is.”*

Although participants agreed that serving size guidance should be publicly disseminated, this advice was viewed as irrelevant by those who didn’t have a medical condition (i.e. obesity, diabetes, renal disease):

Group seven participant: *“Let’s just say if you had high cholesterol you would want more information on the food and stuff but I think that at our age, I don’t think it’s really that valid for us.”*

Group nine participant: *“If you were on a diet you would pay attention to food portion size, and, if you’re not, you just eat whatever you want.”*

Indeed, participants were unanimous in agreeing that some dietary serving size suggestions were *“too small”* (e.g. cereal) and frequently questioned the generalisability of such advice to an entire population with a compilation of varying energy requirements (attributed to age, sex, metabolic rates, and physical activity), sporting needs (e.g. extra protein for building/maintaining muscle mass), and, feeding styles (e.g. meal frequency and structure):

Group seven participant: *“30g of cereal…that’s like, for a guinea pig! But who is a serving for? You don’t know if it’s for a small, petite lady or is it for us lads who play rugby? That can be wrong so how do you judge by that.”*

Group four participant: *“Because I’m eating small I eat more often, probably having five meals.”*

#### ***(2) Guiltless eating***

Discussion within this theme concentrated largely on perceptions about the healthiness of food (i.e. *“if it’s healthy, I can eat more”)* and the prevalent belief that you could eat ‘extra’ after exercise (despite consciously acknowledging that energy consumption may well outweigh energy expenditure). Foremost, it was evident that participants’ beliefs about healthy eating were a major factor in determining ideal portion size, with ‘healthy’ foods being exempt from the practice of portion control. For example, foods such as: chicken, potatoes, cereal, fruits, vegetables, salad, eggs, and pasta, were deemed ‘healthy’ and their portion size control was seen as unnecessary. Conversely, chocolate, cake, biscuits, crisps, ice-cream, cola, sweets, cakes, chips, coleslaw, and processed/take-away food, were classified as ‘unhealthy’ and thus eligible for restriction. For example, one participant stated:

Group seven participant: *“I don’t think portion sizes really matter for the likes of chicken and potatoes and all, but it does for chocolate or a cake, like there’s a cake in my fridge…you’re not going to eat the whole thing. Although some people might, I wouldn’t. For healthy food, you’ll just eat away and away at it cos you know it’s fine…like if I’m making scrambled egg, I’ll just throw 5–6 eggs in it”.*

Various factors influenced the healthy/unhealthy categorisation of foods, such as their perceived: nutritional content and the addition of additives; level of processing; country of origin; and some beliefs related to their advertising (e.g. *“that impression that you’ll fit into the wee red dress”*)*.*When the moderator enquired specifically about the influence of low-fat nutrition claims on consumption, there was an agreement that low-fat invariably meant *“low-taste”*, with most participants stating that they would rather avoid low-fat foods, and *“just eat less”* of the its full-fat counterpart. It was widely acknowledged that low-fat food was not necessarily low in energy due to its typical see-saw relationship with sugar, as illustrated below:

Moderator: “*And if something was labelled as low-fat, does that influence how much you eat?*

Group ten participant: *No, because its low-fat doesn’t mean there’s no sugar in it.*

Moderator: *How about low-sugar labelling?*

Group ten participant: *No, cos then again it could be high in fat. Again it’s turning the packet over and reading, cos just because something says its low-fat, they still have to have flavour in it. So they’ve taken the fat out and put the sugar in it.*”.

#### ***(3) Lack of self-control over food cues***

There was agreement among many participants that the mere presence of large quantities/servings of food, would simply prompt overconsumption as it gave one the liberty to consume more:

Group seven participant: *“Like today I went and got five boxes of Rice Krispies because it was on offer. I’ll eat more. The same goes for your car, if the petrol’s like up here, you go driving all over!”*

Specifically, larger pack sizes (e.g. kingsize and share packs) generally increased consumption, as it was particularly difficult for participants *to “close up the pack”* and *“resist temptation”*. Additionally, single householders felt that a wide range of product packs were often designed for two people, and, in a similar way, also prompted them to overeat:

Group four participant: *“I live on my own so like there are a lot of things that you’d buy are designed for two people. Do you know what I mean? And then, you’d end up eating three-quarters of it or, probably eat the whole pack. Rice for example. They don’t cater for one person.”*

Despite several participants reporting that multi-pack servings would provide a discrete stopping point and form a *“psychological”* barrier to further consumption, others viewed multi-pack servings as *“just too tiny”* and as a result consumed an additional pack to compensate for the reduction in pack size.

#### ***(4) Distracted eating***

Participants recognised that many elements of the eating environment often diverted their attention away from their dietary intake and subsequently resulted in a lack of portion size control. Specifically, participants reported that it was possible for them to show no meal-to-meal compensation for increased energy intakes when larger portions were consumed:

Group four participant: *“But if it’s something you’re doing automatically, then you’ll have your big breakfast, your large lunch, your large dinner and later on you might even order Chinese food.”*

Furthermore, socialising with friends and family (e.g. at parties, restaurants) reportedly obscured the participants’ ability and motivation to monitor food consumption in addition to distractions within the work and home environment (such as televisions, computers and phones), for example:

Group one participant: *“I think too it depends on what you are doing, if you’re sitting at the table and you’re eating, I think you’re more inclined to say that I’m full. Whereas if I’m sitting in the living room, and I’ve a bad habit of that, watching TV and eating, I look down and my plates empty.”*

Group one participant: *“I think as well, if you’re having a night in, like a DVD with friends and bowls of popcorn and sweets, no-one’s thinking about portion sizes. It’s kind of your spoiling yourself you know.”*

#### ***(5) Social pressures***

Despite reportedly self-restricting portion size in the company of certain people (e.g. friends), most participants admitted that they also regularly consumed larger portion sizes in order to impress or ingratiate themselves to hosts/fellow diners. Overall, participants collectively agreed that there was a uniform requirement to be a *“good guest”* when visiting someone’s home. This often involved consuming unappealing food (principally due to taste) to beyond the point of fullness; in order to be polite and not offend the host. One participant stated:

Group seven participant: *“If you’re at somebody’s house and even if it’s a terrible feed like, you’re not going to leave it all, you’re going to eat a certain amount, so they don’t get offended, you know.”*

In some instances, males in particular viewed eating as a *“test of manhood”*, and, described these instances where they were teased by their male peers because of their reluctance to consume more:

Group four participant: *“Let’s say I was out on a Saturday or whatever, with a girl, you’re not going to sit and pig your face out, the way you do at home. You’re going to be half reserved. It’s completely barbaric when its men only, you just go mad. Same as drink, you’re trying to out-drink each other.”*

Group seven participant: *“Or when you’re there like, eating Chinese food and then you don’t feel like anymore. And then there’s a man aspect comes in…someone’s like, ‘finish that you fairy’*!”

Females also reported increasing their habitual portion sizes in the company of male diners to avoid appearing *“mean about food”* or feeling *“like a spoil sport”.* For instance, one participant reported that she didn’t want to give the impression that she was *“constantly counting calories”.*

#### ***(6) Emotional eating rewards***

Within this theme, participants described psychological factors inherent within themselves which impacted on their food portion size consumption. Participants reported an association between emotions and the consumption of larger portions of certain *“comfort”* foods, such as ice-cream, chocolate, and crisps. These *“comfort”* foods reportedly provided a positive elevation in mood when participants were feeling upset, rejected, worried, stressed, bored or depressed:

Group six participant: *“Well if I’m bored, or if I’m having a really bad day, I’m like right give me the chips and dip- I’m eating the whole bag! So whereas if I was in a good mood and I thought I was being good and active on that day, I wouldn’t eat as much because I wouldn’t be sitting there thinking “Right, I need to cheer myself up.”*

#### ***(7) Quantification habits ingrained from childhood***

All participants’ stated that their portion sizes were determined by *“habit”*, with food items repeatedly being prepared, served, and consumed in the same quantities. Frequently, participant’s spontaneously acknowledged that their portion sizes were guided by their mothers’ practices when they were growing up, which, in turn, were often influenced by traditional culture and customs:

Group eight participant: *“But I think that’s [portion sizes] set by even your upbringing, because my husband doesn’t eat so much because he was never given large portions but I would eat more because we were obviously given bigger portions when we were young.”*

Group six participant: *“But I think it’s an Irish thing too. In Ireland we always feed people with potatoes, we eat way too much carbohydrate. You see people putting potato on top of rice!!”*

In addition to embedding portion norms, parents often instilled participants with core values in relation to food waste during their childhood; with the result that many felt obligated to finish all the food on their plate. This was particularly true for the more expensive foodstuffs, such as meat. Many recalled instances whereby their parents used tactics, such as, guilt (*“there are people starving in Africa”*), bribery (“*you won’t get dessert”*) or a battle of wills (*“you can’t leave the table”*) to encourage them to finish all the food on their plates from an early age. In some cases, participants perceived that they were encouraged to eat large portions sizes, not only on account of reducing food waste, but also because they appeared poorly nourished to their mother:

Group six participant: *“Probably the reason why I would actually eat more than any other normal person should eat, was, when I was actually younger, even as a child, and my mother used to say I was very, very poorly looking. So, I was the one who was given the plate with everything bunged unto it and I was told ‘there’s people in Africa starving, don’t waste food’. You see, that’s where it stems from, and I would still do that yet.”*

Even for participants who *“retrained their own mind”* to recognise appropriate portion sizes and utilize effective portion size control strategies to prevent overeating, the concept of ‘cleaning the plate’ was still a constant challenge to overcome. For example:

Group one participant: *“… Like now I don’t overeat but if there was a big plate or something in front of me like I definitely would eat it all because I have that thing like I can’t waste this.”*

### Self-reported portion control behaviors

In addition to describing personal barriers to appropriate food portion size control, several participants had a repertoire of strategies and practices which they employed to control the amount of food that they ate at one time. Specifically, participants described five behavioral strategies in the areas of shopping, storage, cooking, serving, and satiation/satiety (Table 4). For example, a few participants chose to buy foodstuffs which were already packaged into portion controlled sizes (a shopping strategy), while others stored food in such a way that it served to reduce the convenience of bulk purchased items in the household (a storage strategy). It is noteworthy that the majority of these strategies were gleaned from those participants who reported following a special diet at the time of participation in the focus group (i.e. a slimming, n=13; diabetic, n=3; or renal diet, n=1).

**Table 4:** Self-reported consumer strategies for overcoming barriers to appropriate food portion size control

Portion control strategy*	**Typical quote**
Shopping:	
Avoid buying tempting foods for one’s own self	*“I would’ve eaten big portions of bread before and that’s my weakness, so I just don’t buy bread anymore”*
Avoid buying food in larger volumes, sometimes opting for food packaged in single individually sold portions instead	*“I stay away from those big bags and a dozen packets of crisps for 50 pence or whatever. I eat 90% that night”*
	*“If I bought a six pack of crisps I would’ve eaten the six bags, but instead I walk over to the shop, buy two bags and that’s me”*
Buy food already packaged into portion controlled sizes	*“I used to weigh rice, but now I use the bags so not anymore”*
Storage:	
Reduce the convenience of bulk purchased foods e.g. freezing	*“So now, when I buy chops, I separate them into bags, bung them into the freezer, keep one out and cook it, because If I didn’t I would eat the rest!”*
Store appropriate dinner serving sizes in advance	*“I might make a frittata and then I would eat it for a few days, but I’d be quite conscious about cutting that up into portions”*
Cooking:	
Cook an appropriate portion and no more	*“I tend to cook just enough so there isn’t seconds [second servings], because if I cooked too much and there was some left, I’d eat it!”*
Serving:	
Use smaller bowls, plates and spoons	*“I’ve lost four stone and part of that was addressing portion size. It was something as silly as buying a smaller bowl”*
Visualise plate in segments	*“I just divided my plate into imaginary segments…one third is fruit and veg, one third is carb, one third is protein”*
Use object references	*“Grapes would be a handful, and meat, chicken or fish would be the palm of my hand, cheese would be a matchbox, and then I just sort of imagine scoops for everything else- rice or potatoes”*
Use weighing scales or cups for serving sizes that are difficult to gauge (e.g. rice, pasta, cereals, milk)	*“I weigh rice because it’s a fickle thing in that you put it into the pot and it grows to a size that’s three times as big so it’s really difficult…to find out how much rice is going to make a portion. You wouldn’t weigh carrots because you can see them”*
Eye-ball or gauge appropriate serving sizes based on past restrictive measuring	*“We used to measure our rice out and stuff like that but now I just sorta bung it in and go by eye and hope I’ve got it right”*
Satiation and satiety:	
Eat satisfying portions of low energy dense foods e.g. cereals, fruit and vegetables	*“I stick the extra vegetables in because you can get away with the smaller portion of the meat, pasta or whatever. Stick extra vegetables in to bulk it out more and it’s also more filling”*
	*“I have a decent bowl of branflakes every morning to keep me going for the rest of the day…to stop me overeating the rest of the day because I’m starving”*
Drink water	*“I drink more because it’s probably dehydration that’s making you feel hungry and not hunger”*
Eat more slowly or take a break	*“In my old mind, suggested serving sizes were very small, but then I got tips like maybe taking a break or kinda eating slower and you learn that that’s enough food for me”*
Eat regularly	*“I just eat smaller bits throughout the day”*
Know your satiation point	*“I just tried it, I found that was enough for me, just one scoop of pasta”*

*The majority of these strategies were reported by participants who were following a medical or weight loss diet at the time of study participation.

## Discussion

There is a paucity of qualitative data exploring consumers’ views on how food portion size decisions are made. The aim of this present study was to provide an in-depth account of the barriers to appropriate portion size control from a consumer perspective. The rich descriptive data derived in the current study highlight that ill-advised portion size behaviors emanate from various psychological, social and behavioral factors which bypass reflective and deliberative control and converge to constitute significant barriers to appropriate portion size control.

Seven barriers emerged from present focus group data to reveal that, while consumers have been exposed to serving size guidance, its usefulness was questioned by the majority, owing to issues regarding its clarity and relevance. While participants did report that inherently ‘unhealthy’ foods should be restricted, the perceived ‘healthiness’ of some foods generally made portion size restriction unnecessary. Although participants were generally aware of how pre-packed foods influenced consumption, some found it difficult to exert self-control when given the opportunity to consume more, and, constant vigilance was required in order not to over-indulge. Participants also attributed their lack of portion size control to quantitative habits ingrained from childhood, social pressures and emotions.

The first barrier (‘lack of clarity and irrelevance of serving size guidance’) emphasises the clear need for *effective* and *meaningful* serving size guidance [32]. This would necessitate: increasing some suggested serving sizes (e.g. cereal) in line with realistic and typical eating habits in the island of Ireland, and, implementing serving size descriptors, such as a ‘deck of cards’, which are less susceptible to misinterpretation (unlike “a glass”) and which also negate the need to weigh food. Moreover, advice to consumers could be improved if the various industry, healthcare professional and non-governmental organisations worked together to adopt a consistent approach to communicating serving amounts. For example, the reported variability [33] within a food type in the industry on-pack suggested portion sizes could be harmonised to reduce consumer confusion and instil trust. In addition, there may be merit in dispelling the myth that suggested serving sizes are relevant only for the treatment of medical conditions (i.e. obesity, diabetes, renal failure). Where possible, advice should be couched with special consideration for the attributes of individuals (i.e. sex, age, physical activity, eating patterns etc.). Indeed, efforts have recently been made to improve the effectiveness of serving size communication taking account of these factors [34].

Given the role of misperceptions (e.g.*“if it’s healthy I can eat more”*) on portion size decisions, it will also be critical to improve consumer understanding of the body’s energy requirements, including the impact of exercise, and increase knowledge about the nutritional content of food and beverages. One way to overcome ‘guiltless eating’ is to use the nutrition information panel on packaged foods. In this case, an intervention would involve not only education to improve consumer understanding, but also motivation to encourage consumers to look at the label in the first place [35].

Unfortunately, however, even if consumers are informed and have the required skillset to use nutritional information, this does not necessarily translate into desirable behavior [36]. In the current study, behavioral primes such as exposure to food, serving sizes, the size of food packaging, and television viewing, converged to form two barriers to healthy portion size control (namely, a ‘lack of self-control over food cues’ and ‘distracted eating’). Overall, these two barriers highlight the need for consumers to be more vigilant and mindful about monitoring their intake within a food environment that is less than benign with regard to energy intake control. Although there is a lack of research on overcoming unwanted influences such as behavioral primes [37], Wilson and Brekke [38] highlight that people must not only be aware of the potential influence, as demonstrated in the current study, but also have the motivation and ability to correct or avoid it. Indeed, a number of participants (mostly those following a medical or weight loss diet) acknowledged that they used strategies related to the shopping, storing, cooking, serving and satiety of foodstuffs to overcome their self-identified barriers to appropriate food portion size consumption (Table 4). Given the predictive power of self-efficacy [39] on health behavior change, perhaps there is also merit in strengthening people’s personal efficacy beliefs (through *vicarious experiences*) by raising awareness about how consumers in this study are employing portion control strategies (Table 4).

In addition to behavioral primes, participants in this study also identified other unwanted real-world influences which formed two barriers to appropriate portion size control: ‘social pressures’ and ‘emotional eating rewards’. These barriers often prevented consumers’ good intentions from being translated into action. Previous research has highlighted that there may be benefit in forming an ‘if-then’ plan or implementation intention (i.e. *“when situation x arises, I will initiate the goal-directed response of y”* qtd. from [40]) in advance of encountering these situations. Such plans or intentions mean that the goal-directed response occurs automatically when the critical situation arises and does not require conscious intent. Previous research has also highlighted the benefit of using implementation intentions geared at strengthening self-efficacy [41].

The final barrier, ‘quantification habits ingrained from childhood’, highlights that once an eating habit is formed, the behavior is often repeated with little input from reflective processes. This is supported by previous work showing that people often repeat past eating habits, without activating behavioral goals or preferences [42-44]. Overriding a habit requires effort [37], and, the demands of daily life such as time pressure and distraction can often hinder progress [45,46]. The current study showed that consumers’ portion size selection habits were guided both by their mothers’ portion size practices when they were growing up, and, exacerbated by parental views in regard to food waste. This indicates that there may be merit in adopting family-based behavioral/lifestyle counselling to promote appropriate portion size control and to encourage repeated practice of more healthful behaviors from an earlier age. In addition, interventions which encourage portion-control strategy use (like those detailed in Table 4) and change environmental cues to unhealthful portion control may be used to help break established habits [37]. Such environmental opportunities could target cues within the consumers’ physical, economic, political and socio-cultural environment [22], and may include, for example, decreasing the size of foods portions being served in restaurants.

## Conclusions

An important strength of this study was that it employed qualitative methodology to directly assess consumer perceptions about factors influencing their portion size consumption. A few limitations deserve consideration. Firstly, we note that while our recruitment approach captured a range of views, it did not permit us to compare and contrast certain sub-groups in our sample (e.g. we were unable to detect age-related differences in barriers and strategy use). Furthermore, the majority of our participants were highly educated which may limit the transferability of our findings. Despite these limitations, our findings contribute to a greater understanding of the barriers faced by consumers in relation to appropriate portion size control. Portion size control strategies should empower consumers to overcome these barriers, and, encourage repeated practice in appropriate portion size control so that better choices become automatic and habitual. Foremost, serving size guidance needs to resonate with consumers and be seen as relevant to their eating behavior. Interventions should raise awareness of ‘portion size distortion’, correct misconceptions regarding food nutritional properties, and, raise awareness of behavior priming effects, while at the same time advising on the various options to overcome unwanted influences.

## Abbreviations

ROI: Republic of Ireland; NI: Northern Ireland.

## Competing interests

The authors declare that they have no competing interests.

## Authors’ contributions

All authors participated in the design of the study. MS, LEH, SO and LKP were responsible for volunteer recruitment and MS conducted the focus groups. MS and MD reviewed the focus group transcripts and applied thematic codings. LKP also coded three of the transcripts. MS and MD extracted themes from the data codings and drafted the manuscript. All authors critically reviewed and approved the final manuscript submitted for publication.
